# A Novel Experimental Method and Setup to Quantify Evaporation-Induced Foaming Behavior of Polymer Solutions

**DOI:** 10.3390/polym17152025

**Published:** 2025-07-24

**Authors:** Xiaoyi Qiu, Zhaoqi Cui, Ming Zhao, Jie Jiang, Wenze Guo, Ling Zhao, Zhenhao Xi, Weikang Yuan

**Affiliations:** 1 State Key Laboratory of Chemical Engineering and Low-Carbon Technology, East China University of Science and Technology, Shanghai 200237, China; ss-xiaoyi-ss@163.com (X.Q.); cuizhaoqi1998@163.com (Z.C.); zhao_mingzzz@163.com (M.Z.); jiangjie@ecust.edu.cn (J.J.); zhaoling@ecust.edu.cn (L.Z.); wkyuan@ecust.edu.cn (W.Y.); 2 Key Laboratory of Smart Manufacturing in Energy Chemical Process, Ministry of Education, East China University of Science and Technology, Shanghai 200237, China; 3 Shanghai Key Laboratory of Multiphase Materials Chemical Engineering, East China University of Science and Technology, Shanghai 200237, China

**Keywords:** foam stability, evaporation-induced foaming, bubble nucleation, logistic growth function, polymer solution

## Abstract

This study provides a novel experimental setup and methodology for the quantitative investigation of evaporation-induced foaming behaviors in a polymer/small-molecule solution system (PSMS). In traditional dynamic test methods, it is difficult to precisely describe the evaporation-induced foaming process of a multicomponent solution because the concentration of light components in solution continuously decreases during ebullition, causing undesired changes in foaming behavior. In this study, a precisely controlled condensation reflux module was introduced into the setup to maintain pressure, temperature, and concentration of the PSMS at constant levels during the entire ebullition process, allowing dynamic test methods to quantify the evaporation-induced foamability. With this newly proposed device, experimental data of typical PSMS, polyolefin elastomer (POE)/n-hexane solution system, were obtained and modeled to illustrate the foam growth profile, thereby characterizing the dynamic foaming process based on a logistic growth function. The corresponding dimensionless number Σ*evap* was calculated to evaluate evaporation-induced foam stability by analyzing the foam growth profile under varying pressure, concentration, and energy input levels. Furthermore, given that the PSMS represents a highly non-ideal system, the bubble nucleation rate *J* was modified in this work by introducing a correction coefficient δ to account for the non-ideal effects of macromolecules present in solutions. Additionally, another correction coefficient *λ* was incorporated into the Gibbs free energy term to adjust for supersaturation of liquid during nucleation. The experiment’s data align well with the modified bubble nucleation rate mechanism proposed herein.

## 1. Introduction

The phenomenon of evaporation-induced foaming in polymer/small-molecule solution systems (PSMSs) is a significant concern across various industrial applications. In most exothermic polymerization reactors, a substantial portion of reaction heat is removed from the system through an evaporation-condensation cycle [1,2,3]. The formation and growth of foam within the reactor can lead to flooding and entrainment as it ascends toward the condenser tower. Once the polymer foam enters the condenser, it may result in coking and scaling, which will significantly reduce heat transfer performance and increase pressure losses [4,5]. In extreme cases, uncontrolled foaming can precipitate overheating failures in polymerization reactors, posing serious safety issues [6]. Consequently, there has been extensive interest from both industry and academia regarding the foaming behavior of the PSMS at varying concentrations under different pressures and reaction heat powers within polymerization reactors [7,8,9].

Evaporation processes in polymerization reactors involve unique foaming and bubbling behavior due to the distinct thermodynamic properties inherent to PSMSs [10]. The evaporation-induced foams present in PSMSs, consisting of small-molecule gas bubbles dispersed throughout a liquid phase of polymer solution, typically maintain a dynamic equilibrium between the generation and destruction of bubbles, mainly influenced by gas generation volume and liquid film stability [11,12,13]. Gas generation volume is strongly affected by energy input powers for evaporation, where either reaction heat from inside the system or transferred heat from outside the system performs a similar role. Additionally, pressure and temperature also play critical roles in influencing gas generation volume by affecting the density of gas in bubbles. However, temperature cannot be treated as an independent variable in this case since boiling temperature during gas–liquid two-phase processes involving PSMSs depends on both pressure and concentration [14]. Liquid film stability is determined by surface tension and pressure difference between the interior and exterior sides of the liquid film [15]. For a given composition of boiling PSMSs, the surface tension is primarily influenced by boiling temperature [16], which is dependent on both pressure and concentration as described above. In summary, evaporation-induced foaming for a fixed composition of the PSMS represents an equilibrium that can be either stabilized or destabilized based on factors such as energy input power, polymer concentration, and pressure of the solution system.

This research introduces a novel experimental setup and methodology to quantitatively investigate the behaviors of evaporation-induced foaming in polymer solutions. The experimental data obtained from this approach were modeled to create foam growth profiles that describe the dynamic foaming process according to Bikerman’s definition of formability [17]. A dimensionless number ∑evap, proposed by Laura [18], was calculated from these experimental results to evaluate the stability of evaporation-induced foam. Furthermore, the bubble nucleation rate was derived and calibrated from foam growth profiles to validate the bubble nucleation mechanism in PSMSs based on Blander’s theory [19].

## 2. Theoretical Evolution

### 2.1. Foam Growth Profile

Foam is composed of a multitude of gas bubbles dispersed in a liquid phase, where the bubbles continuously generate, grow, and collapse over time. These interrelated processes collectively contribute to the observed foam growth profile. The investigation of foam growth profiles aims to enhance our understanding of foaming behavior and performance under varying conditions. Furthermore, the foam growth profile serves as a fundamental basis for evaluating foam stability and exploring bubble nucleation mechanisms.

Moris [20] proposed an analytical framework based on unit models to examine the dynamics of foam growth. This model indicates that surface tension and initial radius have a low impact in practical applications, while thermodynamic driving forces, unit mass (which is inversely related to bubble density), and mass momentum transfer significantly influence the characteristics of foam growth profiles. In cases involving evaporation-induced foaming within specific PSMSs, thermodynamic driving forces are associated with energy input powers supplied for evaporation. Bubble density and mass momentum transfer correlate with pressure and temperature variations. Notably, boiling temperature is a function of polymer concentration and the pressure of the solution system. Thus, it can be concluded that the energy input powers, pressure conditions, and polymer concentration represent three main factors affecting the foam growth profiles during evaporation-induced foaming in a certain PSMS.

Robert [21] introduced a multi-scale framework for simulating the dynamics of dry foam by encompassing membrane rearrangement, drainage phenomena, and rupture events. This model revealed that the evolution of the foam growth profile consists of both an accelerated growth zone followed by a decelerated growth zone. During the initial stage, the rate of foam growth is relatively slow due to the limited number of bubble nuclei; however, as the foam volume increases—resulting in more bubble nuclei—the foam growth rate accelerates correspondingly. When the volume of the foam layer reaches a critical threshold value, the effect of bubble rupture becomes non-negligible, causing the foam growth rate to slow down, and eventually the foam reaches an equilibrium and stops growing. Based on this theory, the logistic growth function is suitable to describe foam growth profile, and Wu [22] applied it in the low-density polyethylene foam growth process with a high correlation coefficient of 0.967. In this work, we will follow this method and use a logistic growth function to fit the experimental data and to describe the foam growth profile of evaporation-induced foaming in PSMSs as shown in Equation (1),

(1) $$V_{f}\left(t\right)=\frac{V_{f,max}}{1+\exp\left(-k\left(t-t_{c}\right)\right)}$$

where $V_{f}\left(t\right)$ is the foam volume at time t, [m^3^]; $V_{f,max}$ is the maximum of the foam volume, [m^3^]; k is the rate constant for foam growth, [s^−1^]; and $t_{c}$ is the time when the foam reaches its maximum growth rate, [s].

Notably, when $t=t_{c}$, Equation (1) becomes $V_{f}=V_{f,max}/2$ which indicates that the growth rate will begin to decrease once the bubble layer reaches half of its maximum volume.

### 2.2. Evaluation of Foam Stability

To evaluate foam stability, many characterization and measurement methods, including static methods and dynamic methods, have been proposed for different application scenarios. For static methods, a certain amount of foam is pre-generated, and then the time required for foam to be reduced by half (aka. foam half-life t_1/2_) is measured as the drainage rate of the bubble film which can be regarded as an indicator to evaluate foam stability. Bartsch [23] was the original founder of this method, and Miles [24] further popularized it as the Waring Blender method. Many researchers are investigating foamability with this method to screen and modify surfactants in different solution systems [25,26]. Static method is simple and practicable, however, for evaporation-induced foams, it has deficiencies in describing dynamic foam stability, coupling the equilibrium of bubble generation and destruction.

For dynamic methods, a steady-state foam volume is established by balancing the foam generation and destruction under certain conditions in a cylinder tube. The more stable the foam is, the larger the steady volume will be in the tube. Bikerman [17] established the first applicable dynamic method where the test liquid was placed at the bottom of the cylinder tube at a fixed temperature, and gas was introduced into the liquid at a constant flow rate ${V_{g}}^{\prime }$. The foam in the tube would grow and reach an equilibrium volume $V_{f}$. The Bikerman index is proposed as $V_{f}/{V_{g}}^{\prime }$ to evaluate dynamic foam stability. Amankeldi et al. [27] investigated foam stability of surfactant/SiO_2_ composite nanofluids through the dynamic method, and a normalized foam height ($H/H_{0}$) was introduced to measure foam height at different time intervals. Yekeen et al. [28] indicated that foam stability could be determined by plotting the normalized foam height as a function of time. The traditional dynamic method is efficient in describing the foam stability of inert gas-induced foams. However, it is inadequate in evaluating evaporation-induced foams because the boiling gas flow rate is unavailable.

Laura [18] adjusted Bikerman’s method by introducing an electric heater with a certain heat flow to replace the inert gas generator. Thus, the characteristic dimensionless number to quantify evaporation-induced foaming stability was proposed as $\sum_{evap}\;$, which was the ratio of maximum foam growth rate to boiling gas flow rate. But for PSMSs, the concentration of polymer in solution changes during the evaporation, and Laura’s method fails to maintain a constant concentration throughout the process. In this work, a precisely controlled condensation reflux module is added to maintain polymer concentration in the PSMS where $\sum_{evap}\;$, shown in Equation (2), can be used to quantify foam stability.

(2) $$\sum evap=\frac{{V^{\prime }}_{f\cdot max}}{{V_{g}}^{\prime }}$$

where ${V^{\prime }}_{f\cdot max}$ is the maximum of the foam growth rate, [m^3^/s], which can be calculated by derivation of Equation (1); and ${V_{g}}^{\prime }$ is the boiling gas flow rate, [m^3^/s], which can be calculated from the energy power put into the system to evaporate the solvent.

The physical significance of this dimensionless number is the ratio of the amount of gas contained within the bubble to the total amount of gas produced by boiling in per unit time.

### 2.3. Bubble Nucleation Mechanism

According to classical bubble nucleation theory [19], the Gibbs free energy change ∆G for the formation of a bubble within a system is determined by two components: the Gibbs free energy change associated with volume ${∆G}_{vol}$ and the Gibbs free energy change associated with the surface area ${∆G}_{surf}$.

(3) $$∆G={∆G}_{vol}+{∆G}_{surf}$$

(4) $${∆G}_{vol}=-\frac{4}{3}\pi r^{3}\left(P_{in}-P_{out}\right)$$

(5) $${∆G}_{surf}=4\pi r^{2}\sigma$$

where ∆G is the Gibbs free energy change of bubble formation, [J]; ${∆G}_{vol}$ is the Gibbs free energy change associated with volume, [J]; ${∆G}_{surf}$ is the Gibbs free energy change associated with surface area, [J]; r is the radius of the bubble, [m]; $P_{in}$ is the pressure inside the bubble, generally considered as the saturated vapor pressure of pure solvent, [Pa] [19]; $P_{out}$ is the system pressure outside the bubble, [Pa]; and σ is the surface tension of the bubble layer, [N/m]. $R_{cr}$ is the critical radius of a bubble where the Gibbs free energy change equals zero. When the bubble radius is smaller than $R_{cr}$, the Gibbs free energy change ∆G>0, making the bubble unstable and collapse, whereas for $r>R_{cr}$, the Gibbs free energy change becomes negative ∆G<0, driving the bubble to expand and grow.

(6) $$R_{cr}=\frac{2\sigma }{P_{in}-P_{out}}$$

(7) $${∆G}_{cr}=\frac{16\pi \sigma^{3}}{3{\left(P_{in}-P_{out}\right)}^{2}}$$

where $∆G_{cr}$ is the Gibbs free energy of formation in unit, [J].

And the bubble nucleation rate J can be expressed as Equation (8):

(8) $$J={N_{0}\left(\frac{2\sigma }{\pi mB}\right)}^{0.5}\exp\left(-\frac{{∆G}_{cr}}{k_{B}T}\right)$$

where J represents formation rate of bubbles in per unit volume, [pcs/(m^3^·s)]; $N_{0}$ is the number of molecules per unit volume, [pcs/m^3^]; m is the mass of the gas in bubble, [kg]; $k_{B}$ is Boltzmann constant, [J/K]; T is temperature in Kelvin, [K]; and B can be expressed as Equation (9):

(9) $$B=1-\frac{1}{3}\left(1-\frac{P_{out}}{P_{in}}\right)$$

By substituting Equation (7) into Equation (8), we have the equation to describe the bubble nucleation rate for an ideal system, as shown in Equation (10).

(10) $$J=N_{0}{\left(\frac{2\sigma }{\pi mB}\right)}^{0.5}\exp\left(-\frac{16\pi \sigma^{3}}{3k_{B}T{\left(P_{in}-P_{out}\right)}^{2}}\right)$$

However, many researchers have found that this model appears inadequate in describing the high-speed foaming process in polymer solutions [29]. Considering the PSMS is a highly non-ideal system, classical bubble nucleation theories are not fully satisfactory. In this paper, Equation (10) is modified to Equation (11) as a correction coefficient δ was introduced to correct the non-ideal influence of macromolecules in solution, and another correction coefficient λ was added to the Gibbs free energy term to modify supersaturation of liquid on the free energy of the PSMS during nucleation.

(11) $$J=\delta \;N_{0}{\left(\frac{2\sigma }{\pi mB}\right)}^{0.5}\exp\left(-\lambda \frac{16\pi \sigma^{3}}{3k_{B}T{\left(P_{in}-P_{out}\right)}^{2}}\right)$$

## 3. Experiment

### 3.1. Experiment Setup

**Figure 1:**
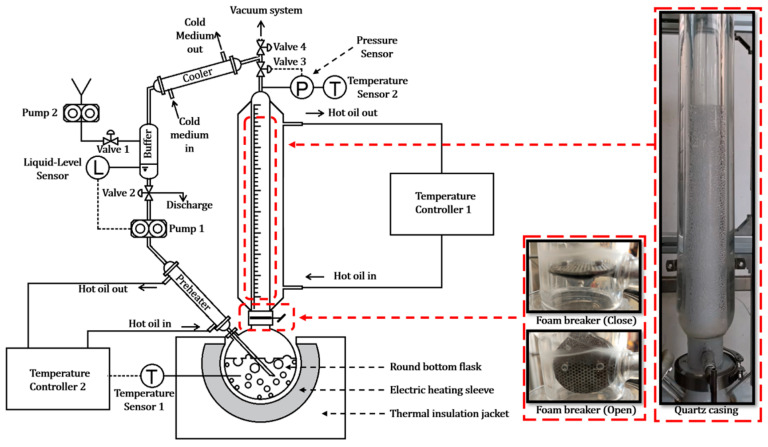
Schematic diagram and photo of the actual experimental setup.

The experimental setup is illustrated in Figure 1. The design was inspired by Laura Strodtman’s apparatus [18], which was primarily intended for studying evaporation-induced foaming behaviors in small-molecule liquids. However, PSMSs differ significantly from small-molecule liquids in their foaming behavior. The concentration of polymer in solution changes with time during the ebullition process, which interferes with the observation of foaming dynamics. To adapt the system for investigating the bubbling and foaming behavior of polymer solutions, we redesigned and modified the original apparatus, allowing it to be capable of maintaining constant concentration during ebullition under both negative and positive pressure conditions. The key improvements are summarized as follows:

(1) Constant Concentration Control: During the vaporization process, the concentration of the polymer solution in the round-bottom flask increases continuously, which significantly alters the foaming behavior. To maintain a constant concentration throughout the experiment, we incorporated a precisely controlled condensation reflux module into the system.
(2) Reheating of Refluxed Liquid: The refluxed liquid must be reheated to near boiling before re-entering the round-bottom flask. If the condensate is too cold, it will absorb a portion of the heating energy from the electric heating sleeve—energy that is intended solely for vaporizing the solution. This would reduce the effective vaporization heat and compromise the experimental accuracy.
(3) Pressure Control: To investigate the influence of pressure on the bubbling and foaming behavior of polymer solutions, we introduced Valve 3, a gas-phase back-pressure regulator, between the quartz casing and the cooler. This valve allows precise adjustment of the upstream pressure, ensuring that the pressure within the round-bottom flask and quartz casing remains stable at the target experimental value.
(4) Foam Layer Dynamics Observation: Studying foam dynamics requires capturing the time-dependent growth of the foam layer. This necessitates initiating observations from a steady state where the system has reached thermal equilibrium, but foam formation has not yet begun. To achieve this, we innovatively installed a foam breaker between the round-bottom flask and the quartz casing. The foam breaker consists of three intricately designed porous sieves with staggered capillary channels. These allow vapor generated in the flask to pass freely, while intercepting foam carried by the vapor. This design enables the establishment of a reflux equilibrium with the foam breaker closed. Once equilibrium is achieved, opening the foam breaker allows immediate observation of the foam entering and growing in the quartz casing.

### 3.2. Experimental Steps

Based on the experimental setup described above, 15 sets of experiments under different conditions were conducted to validate the reliability of the apparatus, as summarized in Table 1. Below, the complete experimental procedure for Exp 8 is outlined as an example.

Step 1: The experimental chemicals include polyolefin elastomer (POE) plastic pellets (purchased from Dow Chemical Midland USA, ENGAGE™ 8150) and analytical-grade n-hexane (purchased from Macklin Shanghai China, analytical grade). A total of 4 g of POE pellets and 196 g of n-hexane were pre-mixed and dissolved in a stirring tank. After cooling to room temperature, the mixture was transferred to a sealed glass jar for later use, resulting in a 2%wt concentration of POE/n-hexane in the PSMS.

Step 2: Approximately 100 mL of pure n-hexane was pumped into the buffer via Pump 2. Half of this amount was transferred into the round-bottom flask via Pump 1. Valve 4, Valve 3, and Valve 1 were fully opened, and the vacuum system was activated to evacuate the entire apparatus. At this stage, n-hexane in both the buffer and round-bottom flask began to boil, displacing the air within the system. After 3 min of displacement, Valve 4 was closed, and the vacuum system was turned off. Valve 3 was set to 70 kPa(a) automatic mode (i.e., the valve remains closed when the pressure sensor reads below 70 kPa(a) and opens when it exceeds 70 kPa(a)). The electric heating sleeve was activated at low power to heat the n-hexane until boiling (recommended setting: 20 kW), and Temperature Controllers 1 and 2 were set to maintain the heat transfer oil at the boiling point of n-hexane at the current pressure (58 °C, or by gradually increasing the temperature until no more n-hexane refluxed on the quartz casing). Meanwhile, the cooling cycle for the cooler was started. As n-hexane continued to vaporize in the system, the pressure indicated by the pressure sensor gradually increased to 70 kPa(a). At this point, Valve 3 opened to vent and automatically stabilized the pressure in the quartz casing at 70 kPa(a). Condensed n-hexane gradually appeared in the cooler, and Valve 2 was kept closed to collect the refluxed condensate into the buffer. This state was maintained until all the n-hexane in the round-bottom flask had evaporated and was condensed back into the buffer. The liquid n-hexane in the buffer was then discharged through Valve 2’s bypass to the outside of the system. At this point, a saturated n-hexane vapor environment at experimental pressure was created in the quartz casing.

Step 3: A total of 150 mL of the 2%wt POE/n-hexane polymer solution prepared in Step 1 was added to the buffer via Pump 2. Valve 1 was closed, and Valve 2 and Pump 1 were opened to transfer the POE/n-hexane polymer solution into the round-bottom flask. Pump 1 was kept running at a high flow rate to ensure that there was no liquid level in the buffer.

Step 4: The foam breaker between the round-bottom flask and quartz casing was closed. The power of the electric heating sleeve was increased to 128 kW, as per the experimental conditions. At this point, the round-bottom flask began to boil vigorously, but the foam was trapped by the foam breaker and remained in the round-bottom flask, while the vaporized n-hexane entered the quartz casing. The opening of Valve 3 increased, and the pressure sensor showed minor fluctuations, but the pressure remained stable around 70 kPa(a). A large amount of condensed n-hexane appeared in the cooler and flowed into the buffer. However, the condensed liquid did not stay in the buffer but was immediately pumped back into the round-bottom flask through the preheater by Pump 1, maintaining an isothermal flow. After some time, when the system stabilized, the temperature controller 2 was set to slightly below the temperature in the round-bottom flask by 2 °C, and automatic control was enabled. At this stage, a steady-state evaporation system was established in the experimental apparatus, with a constant pressure of 70 kPa(a), a constant energy input of 128 kW, and a constant concentration of 2%wt POE/n-hexane polymer solution.

**Figure 2:**
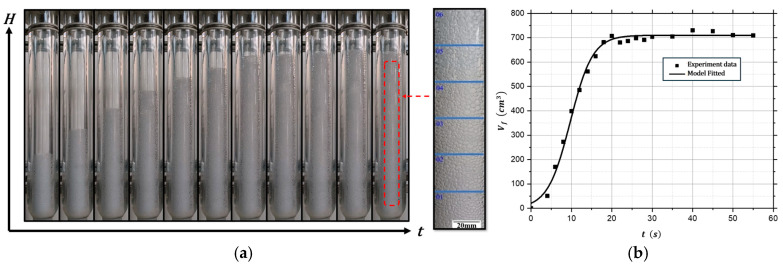
Foam growth profiles for Exp 8 at Pressure: 70 kPa(a), concentration: 2%wt, energy input power: 128 kW: (**a**) foam growth image file, (**b**) foam volume in quartz casing vs. time.

Step 5: The camera was prepared, and the foam breaker was opened. Foam quickly entered the quartz casing and began to rise. The process was recorded with a high-speed camera, as shown in Figure 2a. The moment the foam entered the quartz casing was marked as time zero. The volume curve of the foam layer’s growth and stabilization in the quartz casing was then plotted, as shown in Figure 2b. Quantitative analysis of this curve provided further insights into the foam dynamics.

## 4. Results and Discussion

### 4.1. Foam Growth Profile of POE/n-Hexane PSMS in Different Conditions

**Figure 3:**
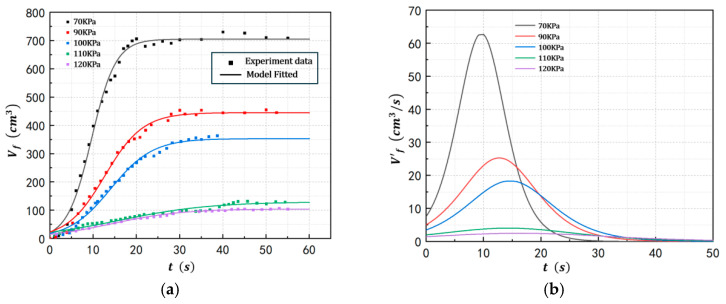
Foam growth profiles at different pressures ((**a**) Bubble layer volume. (**b**) Volume change rate).

To investigate the influence of pressure on foam growth in POE/n-hexane solutions, the evolution behavior of the bubble layer was studied under conditions of a polymer concentration of 2%wt, an energy input power of 128 W, and a pressure range of 70–120 kPa(a). Figure 3 presents the results of foam layer volume and its growth rate as functions of time. It can be observed that lower pressure leads to a higher growth rate of the foam layer, ultimately resulting in a larger stabilized bubble layer volume. For instance, when the pressure is set at 70 kPa(a), the initial volumetric growth rate of the bubble layer is 7.611 cm^3^/s, with a maximum growth rate reaching 62.69 cm^3^/s. In contrast, at a pressure of 120 kPa(a), the initial growth rate is only 1.448 cm^3^/s, and the maximum growth rate is limited to 2.196 cm^3^/s. Furthermore, the final stabilized bubble layer volume at 70 kPa(a) is 705.4 cm^3^, whereas at 120 kPa(a), it is significantly smaller, measuring only 104.7 cm^3^.

The results demonstrate that the pressure significantly influences the foaming capability in POE/n-hexane PSMS. A lower operating pressure (i.e., a greater pressure differential between saturated vapor pressure and operating pressure) effectively enhances gas flow rate by increasing the mass transfer driving force of evaporation and reducing the density of evaporated gas, which significantly promotes the foaming ability. The results and model parameters of Equation (1) for the five runs at different pressures are provided in Table 2.

**Figure 4:**
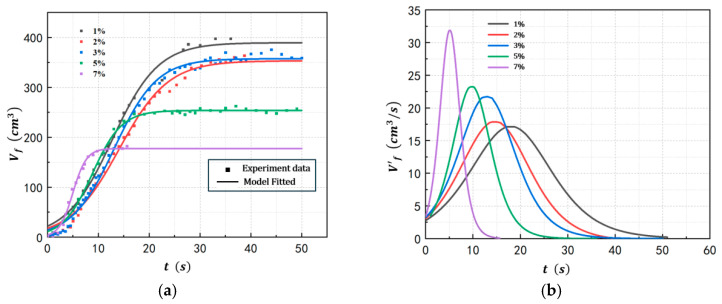
Foam growth profiles at different polymer concentrations ((**a**) Bubble layer volume. (**b**) Volume change rate).

Under controlled operating pressure (100 kPa(a)) and energy input power (128 W), the foam growth profile of POE/n-hexane PSMS was investigated at varying POE concentrations. The experimental results (shown in Figure 4) reveal that increasing concentration enhances the foam growth rate, but it also reduces the maximum volume of the bubble layer $V_{fmax}$. At the concentration of 7%wt, the maximum growth rate of the bubble layer reaches 31.88 cm^3^/s, which is approximately twice that of the 1%wt concentration. However, the volume of the bubble layer at this concentration is only 177 cm^3^, only half that of the 1%wt concentration. The polymer concentration of the solution system primarily affects its viscosity and surface tension. According to Equation (11), the influence of viscosity on bubble nucleation rate is reflected in the parameter δ, while surface tension affects both the Gibbs free energy required for nucleation and the nucleation factor. The increase in concentration increases the solution viscosity, which strengthens the liquid firmness of the foam, making each bubble grow larger and faster, but also decreases the solution saturated vapor pressure, making the maximum volume of the bubble layer shrink. The results and model parameters of Equation (1) for the five runs at different polymer concentrations are provided in Table 3.

**Figure 5:**
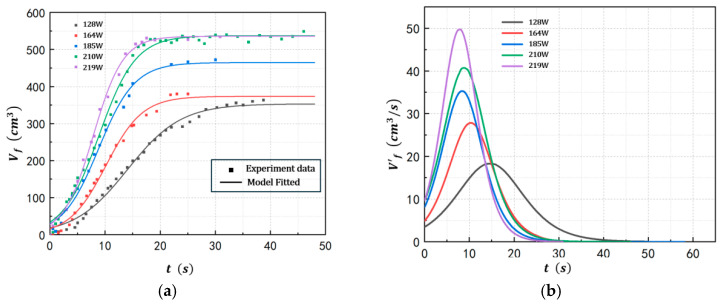
Foam growth profiles at different energy input powers ((**a**) Bubble layer volume. (**b**) Volume change rate).

To investigate the influence of energy input powers on foam growth in POE/n-hexane solutions, the evolution behavior of the bubble layer was studied under conditions of a polymer concentration of 2%wt, a pressure of 100 kPa(a), and energy input powers ranging from 128–219 W, with the results presented in Figure 5. As shown in the figure, higher input power significantly enhances both the volume growth rate and the maximum volume of the foam layer. For instance, at an input power of 219 W, the bubble layer exhibited an initial growth rate of 9.727 cm^3^/s, a maximum growth rate of 49.71 cm^3^/s at time 7.79 s, and a peak bubble layer volume of 504.6 cm^3^. In contrast, at 128 W, these values decreased to 3.513 cm^3^/s (initial growth rate), 18.28 cm^3^/s (maximum growth rate), and 353.4 cm^3^ (maximum volume), respectively. Additionally, elevated input power accelerates the attainment of equilibrium volume, as evidenced by the shorter equilibration time of 27 s at 219 W compared to 45 s at 128 W. That is because larger input power directly increases the amount of gas produced by evaporation, thereby increasing the maximum volume of the foam layer. At the same time, the increase in gas flow rate also accelerates the foam growth rate. The results and model parameters of Equation (1) for the five runs at different energy input powers are provided in Table 4.

### 4.2. Comprehensive Evaluation of Foaming Stability in Different Conditions

As discussed in Section 2.2, the foam stability for the evaporation-induced foaming process in PSMS can be evaluated by the dimensionless number ∑evap. Compared with Equation (1), ∑evap can be reformed as Equation (12)

(12) $$\sum evap=\frac{{\left.\frac{\partial V_{f}}{\partial t}\right|}_{t=t_{c}}}{{V_{g}}^{\prime }}$$

where ${V_{g}}^{\prime }$ is the boiling gas flow rate, [m^3^/s], which can be expressed as Equation (13)

(13) $${V_{g}}^{\prime }=\frac{Q_{r}}{{\;\rho }_{g}H_{r}}$$

where, $\rho_{g}$ is the density of n-hexane gas at experimental conditions, [kg/m^3^]; $H_{r}$ is the corrected latent heat of vaporization of the PSMS, [J/kg]; and $Q_{r}$ is the energy input powers into the system for evaporation, [W].

The study of the foaming characteristic parameters of POE/n-hexane PSMS is based on analyzing the foam growth profile discussed in Section 4.1.

The ratio ∑evap of the two volumetric flow rates, which quantifies the fraction of vapor flow contributing to bubble layer growth, is obtained by applying Equation (12). This parameter can serve as a metric for evaluating the foaming stability of the PSMS. A higher value of ∑evap indicates that small molecules in the solution preferentially transform from liquid into the bubble via foaming, and thus, a more stable foam exists. Conversely, a lower value suggests that small molecules are more likely to transition directly from the liquid phase into the non-bubble gas phase, thereby reducing the stable volume of the foam layer.

The results of ∑evap in different pressures, concentrations, and energy input power are shown in Table 5, Table 6 and Table 7.

As shown in Table 5, the effect of pressure on foam stability is obvious. With the increase in operating pressure, the value of ∑evap decreased by an order of magnitude from 70 kPa(a) to 120 kPa(a). An increase in concentration and energy input power significantly enhances ∑evap, indicating that under high concentration conditions and elevated input power, small molecules are more easily trapped in bubbles and form a foam layer with higher stability.

### 4.3. Bubble Nucleation Rate of POE/N-Hexane PSMS

The foam growth profile obtained in Section 4.1 provides an available approach to quantify the bubble nucleation rate. The entire bubble life cycle is divided into three stages: nucleation, growth, and collapse. The relationship between bubble layer volume and time can be described by the following formula:

(14) $${V^{\prime }}_{f}={V^{\prime }}_{n}+{V^{\prime }}_{g}-{V^{\prime }}_{b}$$

where ${V^{\prime }}_{f}$ is the apparent growth rate of the bubble layer, [m^3^/s]; ${V^{\prime }}_{n}$ is the volume contribution of bubble nucleation, [m^3^/s]; ${V^{\prime }}_{g}$ is the volume contribution of bubble growth, [m^3^/s]; and ${V\prime }_{b}$ is the volume contribution of bubble break, [m^3^/s].

Han’s [30] research demonstrated that during the first few seconds of foaming, the number of bubble nuclei is proportional to time, allowing the foam growth rate to be considered as bubble nucleation rate only. This is because at the very beginning of foaming, there are no mature bubbles in the foam, which means the growth and collapse of bubbles can be neglected and the foam growth rate at 0 time is driven by nucleation only (aka: when t=0, we have ${V^{\prime }}_{f}={V^{\prime }}_{n}$, ${V^{\prime }}_{g}=0$, ${V^{\prime }}_{b}=0$). Thus, the bubble nucleation rate can be expressed as:

(15) $$J=\frac{{V^{\prime }}_{f}\left(0\right)}{\frac{4\pi R_{cr}^{3}}{3}V_{sol}}$$

where ${V\prime }_{f}\left(0\right)$ is the volume change rate of the bubble layer at 0 time, [m^3^/s]; and $V_{sol}$ is the volume of solution, [m^3^].

In Equation (15), ${V^{\prime }}_{f}\left(0\right)$ can be obtained by differentiating the time by the bubble layer volume of the equation; $R_{cr}$ can be obtained by formula (5); and $V_{sol}$ can be measured directly in the experiment.

As discussed above, the bubble nucleation rate under various experimental conditions can be systematically determined using the proposed experimental method through Equation (15), where ${V^{\prime }}_{f}\left(0\right)$ is derived from the experimental results in Section 4.1; and $V_{sol}$ is determined by the amount of the PSMS added into the system. The calculated parameters from foam growth profiles under different conditions are summarized in Table 8.

Using Equation (11), regression fitting was performed on all data, and the model parameters under different conditions were obtained. The results of the model parameters are presented in Table 9.

**Figure 6:**
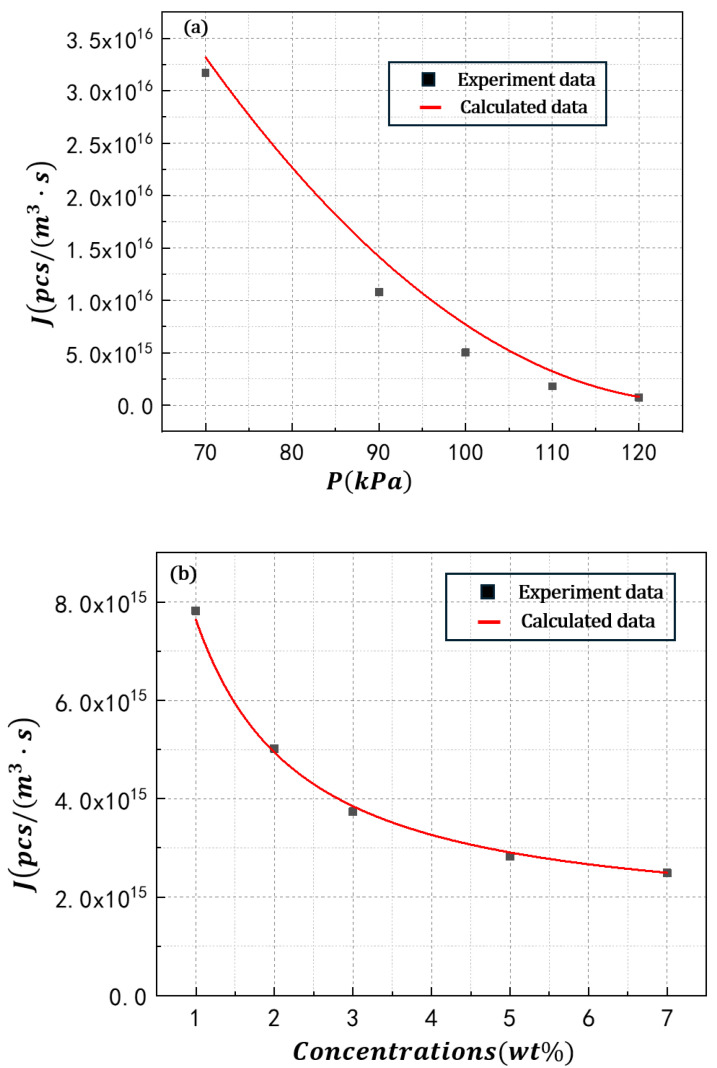

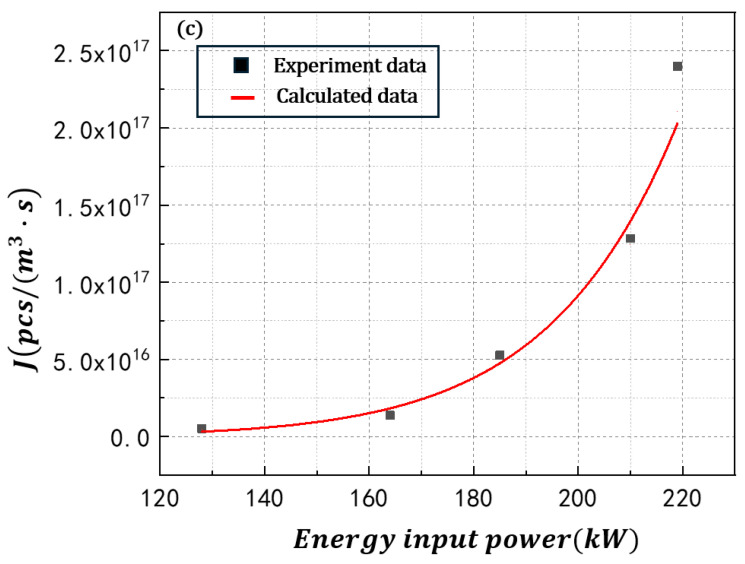
Nucleation rate under different foaming conditions: (**a**) pressure, (**b**) concentration, (**c**) energy input power.

The experimental and regression-predicted bubble nucleation rates of the POE/hexane solution are demonstrated in Figure 6. The results indicate that Equation (11) can effectively describe the bubble nucleation behavior in POE/n-hexane solutions. As illustrated in Figure 6a, the experimental nucleation rate in polymer solutions increases with reduced induction pressure, because decreased induction pressure lowers the Gibbs free energy required for bubble nucleation, thereby facilitating bubble formation in PSMS. Figure 6b demonstrates that the nucleation rate rises with decreasing polymer concentration, which can be attributed to the reduction in surface tension at lower concentrations. This surface tension decrease similarly reduces the energy barrier for bubble nucleation, consequently enhancing the nucleation rate. Figure 6c further reveals that energy input power promotes bubble nucleation rates in polymer solutions. The increased heating power significantly enhances the solution’s supersaturation at constant pressure, enabling more solvent molecules to acquire sufficient energy for phase transition from liquid to vapor and subsequent bubble formation. These results suggest that the experimental data are in good agreement with the bubble nucleation theory and further ensure the efficiency of the experimental method for investigating the bubble nucleation performance of the PSMS.

The bubble nucleation rate model illustrated above can be applied to describe the initial foam growth rate for polymer solutions, which can be extremely useful for many rapid foaming processes, such as bumping of polymer solutions in reactors and micro-foam of elastomers in porous materials.

## 5. Conclusions

In this paper, a novel experiment method was proposed to quantitatively study evaporation-induced foaming behavior in polymer solutions. The foam breaker, reflux assemblies, and some other auxiliary components were introduced into this device to obtain observable stable foam initiation and growth process with constant solution concentration, which is of great importance in studying the dynamic foaming process of polymer solutions. The foam growth profiles under different conditions were obtained through experiments, and the corresponding dimensionless number ∑evap was calculated to evaluate evaporation-induced polymer foam stability. Furthermore, the bubble nucleation rate was derived and modified based on foam growth profiles, which indicates a potential general application of this experimental method in the study of the foaming process.

Specifically, the foaming behavior of POE/n-hexane PSMS was investigated, focusing on the influence of pressure, concentration, and energy input. The results indicate that lowering the pressure increases the bubble nucleation rate by enhancing the pressure gap between the inside and outside of the bubbles, which effectively reduces the Gibbs free energy barrier for bubble formation. Increasing the polymer concentration of POE/n-hexane PSMS also enhances foam stability due to the increased viscosity of the foam liquid film, although it slows down bubble growth. Furthermore, higher energy input promotes evaporation, which significantly accelerates bubble nucleation and evaporation-induced foam stability. These findings may provide insights into controlling and optimizing foam stability and growth in industrial and scientific applications.

## Figures and Tables

**Table 1:** Foam growth experiment conditions.

Nomenclature	Concentration	Pressure	Temperature	Energy Input Power
	(%wt)	(kPa)	(℃)	(kW)
Exp 1	1	100	68.2	128
Exp 2	2	100	68.3	128
Exp 3	3	100	68.4	128
Exp 4	5	100	68.4	128
Exp 5	7	100	68.5	128
Exp 6	2	70	57.5	128
Exp 7	2	90	65.1	128
Exp 8	2	110	71.5	128
Exp 9	2	120	74.4	128
Exp 10	2	100	68.3	164
Exp 11	2	100	68.4	185
Exp 12	2	100	68.4	210
Exp 13	2	100	68.3	219

**Table 2:** Parameters of logistic growth function fit for evaporation-induced foaming growth profile at different pressures.

Nomenclature	P (kPa(a))	$V_{fmax}$ (cm^3^)	${V\prime }_{fmax}$ (cm^3^/s)	k (-)	$t_{c}$ (s)	${V^{\prime }}_{f}(0)$ (cm^3^/s)
Exp 6	70	705.4	62.69	0.358	9.69	7.611
Exp 7	90	445.3	25.29	0.227	12.7	5.057
Exp 2	100	353.4	18.28	0.207	14.1	3.513
Exp 8	110	129.8	4.035	0.124	14.3	2.002
Exp 9	120	104.7	2.196	0.095	16.2	1.448

**Table 3:** Parameters of logistic growth function fit for evaporation-induced foaming growth profile at different concentrations.

Nomenclature	c (w/w)	$V_{fmax}$ (cm^3^)	${V\prime }_{fmax}$ (cm^3^/s)	k	$t_{c}$ (s)	${V^{\prime }}_{f}(0)$ (cm^3^/s)
Exp 1	1%	378.2	17.15	0.223	17.2	2.799
Exp 2	2%	353.4	18.28	0.207	14.1	3.513
Exp 3	3%	336.6	21.75	0.375	13.0	3.521
Exp 4	5%	244.1	23.26	0.397	9.59	3.523
Exp 5	7%	177.3	31.88	0.823	5.22	3.532

**Table 4:** Parameters of logistic growth function fit for evaporation-induced foaming growth profile at different energy input powers.

Nomenclature	$Q_{r}$ (W)	$V_{fmax}$ (cm^3^)	${V\prime }_{fmax}$ (cm^3^/s)	k	$t_{c}$ (s)	${V^{\prime }}_{f}(0)$ (cm^3^/s)
Exp 2	128	353.4	18.28	0.207	14.1	3.513
Exp 10	164	357.7	27.83	0.294	10.3	4.945
Exp 11	185	407.3	35.30	0.326	8.37	8.219
Exp 12	210	500.8	40.73	0.304	8.85	9.712
Exp 13	219	504.6	49.71	0.375	7.79	9.727

**Table 5:** The results of ∑evap in different pressures.

Nomenclature	P (kPa(a))	${V_{g}}^{\prime }$ (cm^3^/s)	${V\prime }_{fmax}$ (cm^3^/s)	∑evap
Exp 6	70	184.34	62.69	0.340
Exp 7	90	146.69	25.29	0.172
Exp 2	100	133.33	18.28	0.137
Exp 8	110	122.30	4.035	0.033
Exp 9	120	113.04	2.196	0.019

**Table 6:** The results of ∑evap in different concentrations.

Nomenclature	c (w/w)	${V_{g}}^{\prime }$ (cm^3^/s)	${V\prime }_{fmax}$ (cm^3^/s)	∑evap
Exp 1	1%	133.33	17.15	0.129
Exp 2	2%	136.58	18.28	0.134
Exp 3	3%	139.13	21.75	0.156
Exp 4	5%	144.39	23.26	0.161
Exp 5	7%	149.76	31.88	0.213

**Table 7:** The results of ∑evap in different energy input powers.

Nomenclature	$Q_{r}$ (W)	${V_{g}}^{\prime }$ (cm^3^/s)	${V\prime }_{fmax}$ (cm^3^/s)	∑evap
Exp 2	128	133.33	18.28	0.137
Exp 10	164	171.43	27.83	0.162
Exp 11	185	193.14	35.30	0.183
Exp 12	210	219.42	40.73	0.186
Exp 13	219	228.57	49.71	0.217

**Table 8:** Key parameters of bubble nucleation rate calculation in POE/ n-hexane solution.

Nomenclature	${V^{\prime }}_{f}(0)$ (cm^3^/s)	$R_{cr}$ (um)
Exp 1	2.799	1.02
Exp 2	3.513	1.28
Exp 3	3.521	1.41
Exp 4	3.523	1.55
Exp 5	3.532	1.62
Exp 6	7.611	0.89
Exp 7	5.057	1.12
Exp 8	2.002	1.49
Exp 9	1.448	1.79
Exp 10	4.945	1.02
Exp 11	8.219	0.77
Exp 12	9.712	0.61
Exp 13	9.727	0.49

**Table 9:** The model parameter of bubble nucleation rate in POE/n-hexane solutions determined by Equation (11).

Variables	$\delta N_{0}$ (No./m^3^)	λ
Pressure	8.20 × 10^8^	2.85 × 10^−8^
Concentration	1.41 × 10^8^	7.04 × 10^−9^
Energy input power	1.33 × 10^9^	4.44 × 10^−8^

## Data Availability

The original contributions presented in this study are included in the article. Further inquiries can be directed to the corresponding author.
